# Non-Destructive Classification of Organic and Conventional Hens’ Eggs Using Near-Infrared Hyperspectral Imaging

**DOI:** 10.3390/foods12132519

**Published:** 2023-06-28

**Authors:** Woranitta Sahachairungrueng, Anthony Keith Thompson, Anupun Terdwongworakul, Sontisuk Teerachaichayut

**Affiliations:** 1 Department of Food Science, School of Food-Industry, King Mongkut’s Institute of Technology Ladkrabang, Chalongkrung Road, Ladkrabang, Bangkok 10520, Thailand; woranitta.s@gmail.com; 2 Department of Postharvest Technology, Cranfield University, College Road, Cranfield, Bedford MK43 0AL, UK; keiththompson28@yahoo.com; 3 Department of Agricultural Engineering, Faculty of Engineering at Kamphaeng Saen, Kasetsart University, Kamphaeng Saen, Nakhon Pathom 73140, Thailand; fengant@ku.ac.th; 4 Department of Food Process Engineering, School of Food-Industry, King Mongkut’s Institute of Technology Ladkrabang, Chalongkrung Road, Ladkrabang, Bangkok 10520, Thailand

**Keywords:** discrimination, authentication, absorbance spectra, physiochemical properties

## Abstract

Eggs that are produced using organic methods retail at higher prices than those produced using conventional methods, but they cannot be differentiated reliably using visual methods. Eggs can therefore be fraudulently mislabeled in order to increase their wholesale and retail prices. The objective of this research was therefore to test near-infrared hyperspectral imaging (NIR-HSI) to identify whether an egg has been produced using organic or conventional methods. A total of 210 organic and 210 conventional fresh eggs were individually scanned using NIR-HSI to obtain absorbance spectra for discrimination analysis. The physical properties of each egg were also measured non-destructively in order to analyze the performance of discrimination compared with those of the NIR-HSI spectral data. Principal component analysis (PCA) showed variation for PC1 and PC2 of 57% and 23% and 94% and 4% based on physical properties and the spectral data, respectively. The best results of the classification using NIR-HSI spectral data obtained an accuracy of 96.03% and an error rate of 3.97% via partial least squares–discriminant analysis (PLS-DA), indicating the possibility that NIR-HSI could be successfully used to rapidly, reliably, and non-destructively differentiate between eggs that had been produced using organic methods from eggs that had been produced using conventional methods.

## 1. Introduction

Hens’ eggs are among the most popular foods eaten worldwide [1], with a global production of around 1642 billion eggs in 2020 [2] and an average consumption of around 161 eggs per person per year [3]. Hens’ eggs are highly nutritional, containing high levels of protein, and are a rich source of vitamins and minerals, including vitamins A, B12, K and D and Fe, Se, and folate [4,5,6]. The diet on which hens are fed can affect the nutrient levels in their eggs. Hens that are fed on an organic diet have different requirements in rearing from conventional laying hens, mainly related to housing systems and access to outdoor areas. Organic production is also associated with free range, especially on pasture that has been cultivated organically [7]. The costs of producing organic eggs are high, which can result in doubling their retail price, depending on the production process [8]. Raw feed materials must be ingredients that comply with the principles of organic agriculture. In addition, hens must have access to enough clean water, and their diets must be free of antibiotics and hormones [9]. This contrasts with conventional cage-raising systems that usually mean that they are raised in indoor housing and will not be released outside the housing. For feed, hens in conventional production systems receive enough processed layer feed in order to ensure rapid growth and clean water as well as antibiotics and hormones [10,11]. Although chemicals such as antibiotics can be used to prevent and treat poultry diseases and to promote their growth, some farmers use antibiotics inappropriately, which may result in antibiotic residues in tissues, organs, and poultry products [12,13]. These residues can remain in their eggs and accumulate in the consumer’s body, resulting in alterations in the consumer’s microflora, disease, and the development of resistant strains that can cause the body not to respond to antibiotic medicines. Thus, residual antibiotics in the product can be harmful to the health of consumers [11,12]. Ebied et al. [14] examined the residues of antibiotics in organic and conventional eggs and found no antibiotic residues in organic eggs, while detecting oxytetracycline and tylosin residues in conventionally produced eggs at a ratio of 1.6% and 2.4%, respectively, in the total of 125 conventionally produced eggs that were sampled. 

There is some evidence that eggs produced organically contain some chemicals that are different from those from conventionally produced eggs. Florkiewicz et al. [15] showed that the content of sodium and potassium in the albumen, yolk, and whole egg was significantly higher (*p* ≤ 0.05) in hens’ eggs raised organically compared to the eggs of hens reared in cages. Similar results were reported by Banaszewska et al. [16]. Minelli et al. [17] evaluated the chemical properties of organic and conventional hens’ eggs and found that organic eggs had higher protein and cholesterol levels than conventional hens’ eggs. However, Heflin et al. [18] showed that the mineral content of eggs differed with the strain of the hens as well as their age and rearing environment.

There is considerable evidence that people are taking increasing interest in their healthcare and will pay more for organic products. When eggs are marketed, there is no way for consumers to tell whether they have been produced conventionally or organically. It was reported [19] that there were some poultry farms in Germany that labeled their hens’ eggs as “organic”, but production at these farms was not up to the standards of organic agriculture. In addition to defrauding consumers, this also affects product manufacturers, who comply with the production of organic standards, which affects their reputation and consumer confidence.

Several methods have been reported, successfully describing the possibility of discrimination between eggs that have been produced from hens reared on an organic system compared to hens reared on a non-organic system, but those techniques are destructive, time-consuming, complicated, and costly. For example, Campmaio et al. [1] categorized organic and inorganic eggs using HPLC-UV fingerprints. Ruth et al. [20] investigated the carotenoid profile in egg yolks in order to differentiate organic and conventional eggs using HPLC. Tres et al. [21] identified differences between organic and conventional eggs by determining the fatty acid profiles of their yolks using gas chromatography. Therefore, there is a clear need for a simple, reliable, non-destructive method for testing eggs as they are being marketed, in order to determine whether they have been produced conventionally or organically.

Methods that have been shown to provide reliable, non-destructive methods of testing food products include near-infrared hyperspectral imaging (NIR-HSI) and near-infrared spectroscopy (NIRS). These methods can be used to provide different information; for example, NIR-HSI provides spectral and spatial data of an image simultaneously in a single system, while NIRS only provides spectral information at the point of measurement. NIR-HSI and NIRS have been used to non-destructively test quality factors in many products. NIR-HSI has been shown to have good potential for use in predicting the quality of agriculture and food products for non-destructive quality assurance methods [22,23]. 

Several publications have shown that HSI can be successfully used for assessing the quality of eggs, including inspecting for defects and freshness [24], detecting several internal and external qualities [25], predicting their freshness [26], and predicting the S-ovalbumin content to indicate their freshness [27]. HSI has also been successfully used to non-destructively test many other food products, including Xu et al. [28] for discrimination between organic and conventional salmon fillets; Teerachaichayut and Ho [29] for predicting total soluble solids, titratable acidity and the maturity index of limes; Lei et al. [30] for classifying Cheddar cheeses from different brands; Cruz-Tirado et al. [31] for categorizing cocoa bean hybrids to authenticate species; Rios-Reina et al. [32] for determining the origin of pine nuts; Khamsopha et al. [33] for predicted adulteration in tapioca starch; Sricharoonratana et al. [34] to determine the shelf life of cakes; Sahachairungrueng and Teerachaichayut [35] to assess the quality of longans; Sahachairungrueng et al. [36] to assess the relative proportions of Robusta and Arabica beans in roasted ground coffee; and Tantinantrakun et al. [37] for predicting the maturity index of intact pineapples.

These research reports indicate that NIR-HSI has been effectively used in many applications for various agricultural and food products. Therefore, NIR-HSI was tested to determine whether it could be successfully used to discriminate between organically and conventionally produced eggs in order to enhance the confidence of purchasing by manufacturers. In this way, NIR-HSI could be used to guarantee the authenticity of organic hens’ eggs, which would be satisfactory for consumers as well. 

## 2. Materials and Methods

### 2.1. Hens’ Egg Samples

Eggs from ‘Rhode Island Red’ chickens, which had been reared using the conventional cage system, were purchased from a poultry farm in Bangkok, Thailand. This conventional cage system used conventional cages that provided 450 cm^2^ space per bird. Eggs from ‘Rhode Island Red’ chickens were also purchased from a poultry farm in Nakhon Pathom, Thailand, which used an organic system. This farm had been certified by the Department of Livestock Development Organic, Thailand, and the National Bureau of Agricultural Commodity and Food Standards, Thailand. 

All the eggs from both farms had been freshly laid, were of good appearance, and were within the size grade number 5 (45–50 g) [38]. Due to the fact that the quality of eggs can change during storage, the two types of eggs that were purchased were divided based on storage period in order to obtain quality variation in the samples. The eggs (N = 420) of two types were individually divided into 14 groups. Each group contained 20 eggs based on storage time in days, which started from day 1 to day 14. The samples were kept in an air-conditioned room at 25 °C for about 24 h before they were individually measured. After storage, each egg was scanned on both sides in the NIR-HSI by turning it 180° after the first scan. The average spectra of the two measurements was used for the analysis.

### 2.2. NIR-HSI

**Figure 1:**
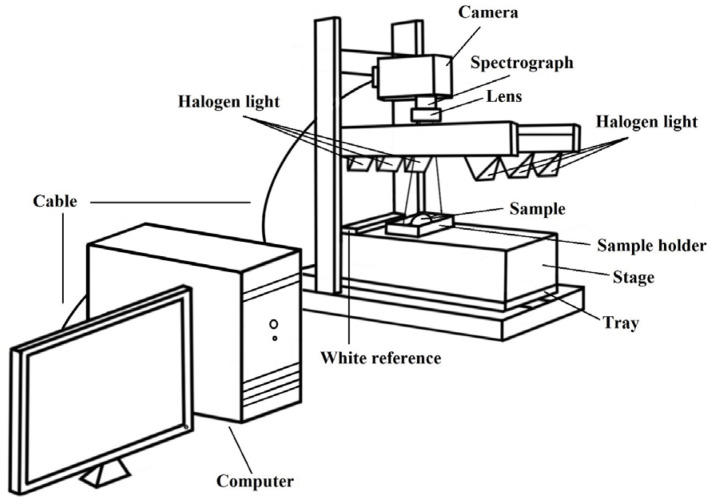
Schematic drawing of NIR-HSI and sample presentation.

**Figure 2:**
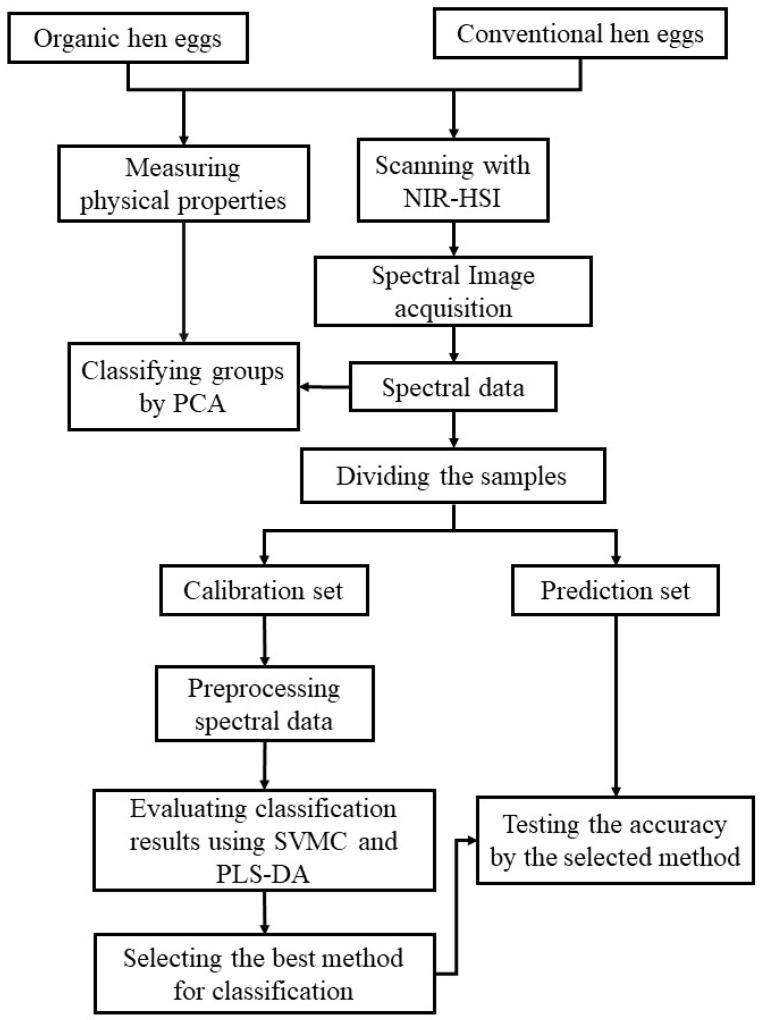
Methodology of classification analysis.

NIR-HSI equipment (Specim FX17e, Spectral Imaging Ltd., Oulu, Finland) was used for the measurements with 224 spectral bands acquired from 935 to 1720 nm with a spectral interval of 3.5 nm and an integration time of 5 ms. Each egg was placed horizontally on the sample holder (the holder had been specifically designed to hold the egg in place and prevent it rolling during measurement), and then, it was transported on a moving tray at a speed of 15 mm/s. There were six halogen lamps (100 W and 12 V each) installed on both sides of the sample, with three halogen lamps lighting each sample at an angle of 45^o^ to the sample, as shown in Figure 1. The image of a dark reference was obtained when the light source was turned off and the lens was covered with a black cap. The image of a white reference was obtained from a white Spectralon tile. The image of each egg was taken from each side (a front side and a back side), and the average spectral image of each sample was used for the analysis (Figure 2).

### 2.3. Physical Properties Measured Using Non-Destructive Methods

#### 2.3.1. Density Determination

The density of each egg was determined using the method outlined by Mohsenin [39], by first weighing each egg in air and then weighing it in water using a scale (Sartorius AG, Göttingen, Germany). The mass of displaced water with the same volume of each sample was determined by weighing the egg when it was submerged in water, and then, the specific gravity of each egg was calculated using the following formula (Equation (1)):

(1) $$\mathrm{Specific}\;\mathrm{gravity}\;\mathrm{of}\;\mathrm{hen}\;\mathrm{egg}=(\frac{\mathrm{mass}\;\mathrm{of}\;\mathrm{whole}\;\mathrm{hen}\;\mathrm{egg}\;\left(g\right)\times \;\mathrm{specific}\;\mathrm{gravity}\;\mathrm{of}\;\mathrm{water}}{\mathrm{mass}\;\mathrm{of}\;\mathrm{displaced}\;\mathrm{water}\;\left(g\right)})$$

The mass of displaced water is M_2_ − M_1_, where M_2_ is the mass of the container, water, and egg, and M_1_ is the mass of the container and water. The specific gravity of water is defined as 1. The density of each egg was calculated according to the following formula (Equation (2)):

(2) $$\mathrm{Density}\;\mathrm{of}\;\mathrm{hen}\;\mathrm{egg}\;(g/{\mathrm{cm}}^{3})=\frac{\mathrm{Specific}\;\mathrm{gravity}\;\mathrm{of}\;\mathrm{hen}\;\mathrm{egg}\;}{\mathrm{density}\;\mathrm{of}\;\mathrm{water}\;\left(g/{\mathrm{cm}}^{3}\right)}$$

where the density of water is defined as 1 g/cm^3^. So, the density of the egg is equal to the specific gravity of the egg.

#### 2.3.2. Shell Color Measurement

The color of each egg shell was determined using a colorimeter (Konica Minolta CR-400, Japan) in terms of its L* a* b*, where L* indicates lightness (with 0 representing no reflection (black) and 100 representing high reflection (white), a* indicates red/green (a positive a* value indicates red and a negative value indicates green), and b* indicates yellow/blue (a positive b* value indicates yellow and a negative value indicates blue)). The shell of each egg was measured at three points in the equatorial region of its surface by turning it every 120°. Average values of L* a* b* were used in the analyses. Before measuring, the colorimeter was calibrated using a white plate.

### 2.4. Physiochemical Properties Measured Using Destructive Methods

The destructive methods of measurements of color and pH were selected instead of other more complicated methods because these techniques were simple, fast, and no chemicals were required. For the measurements, each egg was broken, and its yolk was separated from its albumen, and each was weighed separately. Then, the L* a* b* values of each yolk and albumen were measured as described above.

The pH values of the yolk and the albumen were measured separately using a digital pH meter (Mettler Toledo Seven CompactTM pH/Ion meter S220, Greifensee, Switzerland). Before beginning the measurement, the pH meter was calibrated with a buffer solution at pH 4 and 7.

### 2.5. Data Analysis

The absorption spectra were calculated from the acquisition spectra using the formula (Equation (3)) given by Kleinebecker et al. [40], and the absorbance spectra were used for analysis.

(3) $$A=log\;(\frac{1}{R})$$

where A is the absorption spectra and R is the reflectance spectra.

The acquired spectral image of each egg as well as the background was obtained by scanning using NIR-HSI. The background image was removed from the image, leaving only the sample image, which was then used for analysis. The average spectrum of the sample image was used as the representative of each sample for both organically and conventionally produced eggs. 

The physical properties and physiochemical properties indicated above were determined on each egg. All these variables for each egg were plotted and statistically analyzed using ANOVA, and where significantly different, a *t*-test was performed to determine specific differences between treatments. The results were expressed as a *p*-value where *p* < 0.05 was considered statistically significant.

PCA was performed in order to classify the variables determined from the non-destructive measurements of physical properties as well as the spectral data.

Linear discriminant analysis (LDA) is a statistical technique that is used for classification by separating classes of samples using the classification model that is created by less independent variables. It is used to find a linear combination of features which can differentiate two groups [41,42]. However, for more independent variables, support vector machine classification (SVMC) is a statistical technique where a set of supervised learning methods is used for the classification, regression, and the detection of outliers. It is used to help and to solve two group classification problems [43,44]. Also, PLS-DA is a linear differentiation technique that combines the properties of partial least squares regression with the discrimination presentation of a differentiation technique [45,46].

Physical properties being the independent variables were used for classification analysis using LDA. Also, the average spectral data in the wavelength range of 935–1720 nm were used for classification analysis via SVMC and PLS-DA. In order to determine the optimum classification model for both the organically and conventionally produced eggs, the spectra data were preprocessed by inspecting the results of cross-validation. The pretreatment methods used were smoothing, 1st derivative, 2nd derivative, standard normal variate transformation, multiplicative scatter correction, and combined methods.

The production system of the eggs was the dependent variable. The samples were then arranged into 2 groups: group 0 was organic eggs, and group 1 was conventional eggs.

All 420 eggs in the experiment were divided into two sets: one for regression, called the calibration set containing 294 eggs, and the other for testing the accuracy, called the prediction set containing 126 eggs.

The performance of the classification was evaluated for accuracy (Equation (4)), specificity (Equation (5)), sensitivity (Equation (6)), and error rate (Equation (7)) in both the calibration groups and prediction groups. Here, the accuracy refers to the ability of a group to be classified correctly. The error rate refers to the value of the classification error. The accuracy and error rate are important for evaluating the best classification. Sensitivity describes the capability to memorize the samples of the targeted group. Specificity represents the capability to refuse the samples of the non-targeted group [47]. The formulae used for these calculations were as follows:

(4) $$\mathrm{Accuracy}\;(\%)=\frac{\left(TP+TN\right)}{\left(TP+TN+FP+FN\right)}\times 100$$

(5) $$\mathrm{Specificity}\;(\%)=\frac{TN}{\left(TN+FP\right)}\times 100$$

(6) $$\mathrm{Sensitivity}\;(\%)=\frac{TP}{\left(TP+FN\right)\;}\times 100$$

(7) $$\mathrm{Error}\;\mathrm{rate}\;(\%)=\frac{\left(FP+FN\right)}{\left(TP+TN+FP+FN\right)}\times 100$$

where TN is the true negative samples, TP is the true positive samples, FN is the false negative samples, and FP is the false positive samples.

The IBM SPSS Statistics program (SPSS version 28.0, SPSS Inc., Chicago, IL, USA), Unscrambler X software (The Unscrambler X version 10.4, CAMO Software AS., Oslo, Norway), and Prediktera Evince software (Prediktera Evince version 2.7.9, Prediktera AB, Umea, Sweden) were used for statistical analyses.

## 3. Results and Discussion

### 3.1. Determining Physical Properties via Non-Destructive Methods

**Figure 3:**
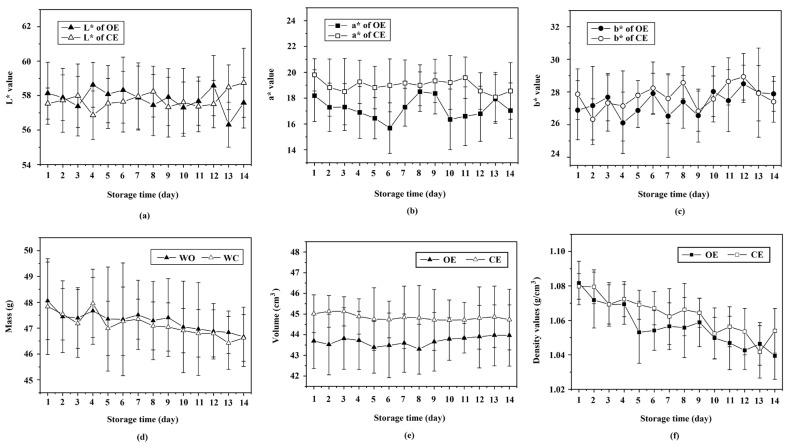
Physical properties of organically (OE) and conventionally (CE) produced hens’ eggs versus storage times: (**a**) L* value, (**b**) a* value, (**c**) b* value of the egg shell, (**d**) mass of whole eggs, (**e**) volume, and (**f**) density.

The aim of this study was to determine whether non-destructive measurements of physical properties of eggs and spectral information were comparable. Therefore, only the physical properties of eggs in this study, which had been determined without cracking the eggs, were used to compare the classification of organic and conventional hens’ eggs with spectral data from NIR-HSI. The lightness (L* value), redness (a* value), and yellowness (b* value) of the shells from the different production methods were found to be significantly different (*p* < 0.05) in that the shell color of conventional eggs had more redness than that of organic eggs, but in practice, this difference was difficult to distinguish visually. The storage time of eggs produced from either the conventional or organic system showed no significant effect (*p* ≥ 0.05) on the L* value (Figure 3a), a* value (Figure 3b), and b* value (Figure 3c), which corresponds to the result of Sokolowicz et al. [48]. Also, the production method did not significantly affect (*p* ≥ 0.05) the mass of the eggs, but the mass of both the conventionally and organically produced eggs significantly decreased (*p* < 0.05) with increasing storage time. This finding agrees with Eke et al. [49] who reported that the egg shell became more porous during storage time, which in turn resulted in weight loss (Figure 3d). The volume of eggs from both production systems did not significantly change (*p* ≥ 0.05) by increasing storage time (Figure 3e), but the volume of conventionally produced eggs was significantly higher (*p* < 0.05) than the volume of organically produced eggs. The density of eggs significantly decreased (*p* < 0.05) during storage, as previously reported by Brodacki et al. [50], but eggs produced using conventional methods were significantly denser (*p* < 0.05) than the eggs produced using organic methods (Figure 3f).

### 3.2. Determining Physiochemical Properties via Destructive Methods

**Figure 4:**
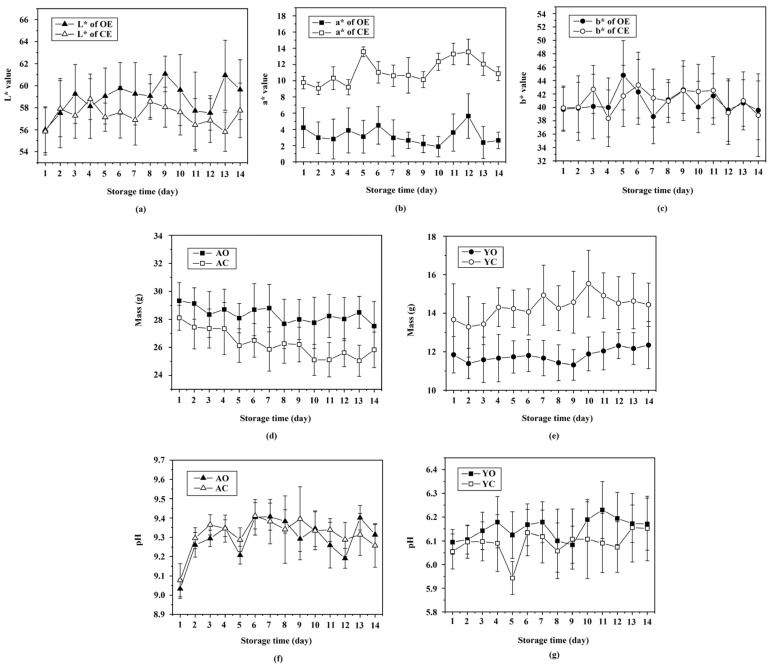
Physiochemical properties of organic (OE) and conventional (CE) hen eggs versus storage time regarding (**a**) L* value, (**b**) a* value, (**c**) b* value of yolk, (**d**) mass of albumen, (**e**) mass of yolk, (**f**) pH of albumen, and (**g**) pH of yolk: AO = albumen of organic hen eggs; AC = albumen of conventional hen eggs; YO = yolk of organic hen eggs; YC = yolk of conventional hen eggs.

The results of the physicochemical analyses of the internal properties of the eggs clearly showed the differences between both types of organically and conventionally produced eggs, but the eggs had to be cracked to determine their physicochemical properties. The L* and a* values of the yolk of both production types were significantly different (*p* < 0.05) (Figure 4a,b), while the b* value showed no significant difference (*p* ≥ 0.05) (Figure 4c). These effects support the report by Lordelo et al. [51] that the color of egg yolks from hens kept in cage systems was darker than that of yolks from hens kept in organic systems. Also, Minelli et al. [17] reported that the lighter yolk color of organic eggs was mainly due to feed factors. Storage time significantly affected (*p* < 0.05) the yolk color of both types, which supports the findings of Jin et al. [52], who reported changes in yolk color after storage for only 2 days. The mass of the albumen of organically produced eggs was significantly higher (*p* < 0.05) than that of conventionally produced eggs, while the mass of the yolk of conventional eggs was significantly higher (*p* < 0.05) than that of the organic eggs (Figure 4d,e). These findings support those of Zotte et al. [53], who showed that organic hens’ eggs had a higher albumen mass and a lower yolk mass than conventionally produced hens’ eggs. Also, Lordelo et al. [51] found that the eggs produced by hens in a cage-rearing system had more yolk than the eggs from hens in an organic system. Also, the albumen mass significantly decreased (*p* < 0.05), while the yolk mass was significantly increased (*p* < 0.05) during storage, which confirms the results of Brodacki et al. [50], who reported that the water content increased in the yolk and decreased in the albumen due to the diffusion of water from the albumen to yolk. There were no significant differences (*p* ≥ 0.05) in the pH of the albumen and yolk between organically and conventionally produced eggs. The albumen and yolk pH values significantly increased (*p* < 0.05) slightly during storage, which supports the results of Lee et al. [54] (Figure 4f,g).

### 3.3. Absorbance Spectra

**Figure 5:**
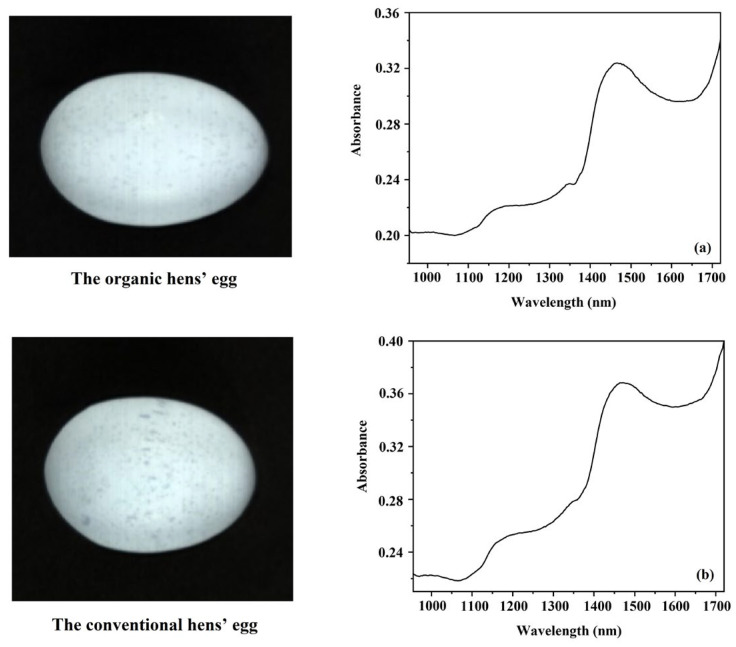
Samples of eggs from the experiment: (**a**) an organic hen’s egg and (**b**) a conventional hen’s egg.

**Figure 6:**
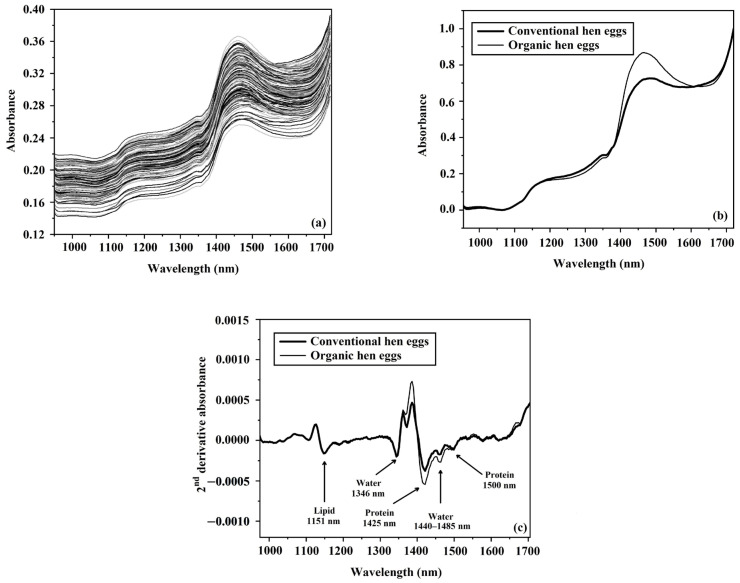
Spectral data of organic and conventional eggs: (**a**) original absorbance spectra of all samples, (**b**) average absorbance spectra scaled via normalization, (**c**) and average 2nd-derivative absorbance spectra.

The curvature of the shell of eggs can affect the way light is reflected from their surface. In order to take this reflectance into account, the position of the sample and lamps was adjusted to obtain the best measurement conditions before starting the scanning of the samples of both the organic and conventional eggs. In Figure 5a,b, the features of eggs and the average spectra of samples from each type of egg are presented. The original spectra of the eggs from all the samples are shown in Figure 6a, and the average spectra for both types are normalized and presented in Figure 6b. The main peak for both types of egg was around 1440–1485 nm (O-H stretch first overtone), which supports the findings of Workman Weyer [55]. The original spectra clearly showed the main peak of water was around 1440–1485 nm, indicating that water is the main component of eggs, which has previously been shown by many researchers [56].

The original average spectra were preprocessed using the second-derivative pretreatment method in order to differentiate the peaks of the chemical composition of the eggs. This showed that there were peaks at around 1151, 1346, 1360, 1425, 1440–1485, and 1500 nm in the second-derivative absorbance spectra of both types of egg production systems (Figure 6c). The peak at around 1151 nm was shown to be associated with C-H stretching overtones related to lipids, and the peak at around 1360 nm was shown to be directly related to lipids [57,58]. Muncan and Tsenkova [59] and Workman and Weyer [55] previously showed that the 1346 nm absorption band was linked to the C-H stretch second overtone of O-H and the 1440–1485 nm absorption bands were linked to the O-H stretch first overtone in water, as discussed above. The absorption peaks at 1425 (O-H and N-H stretch first overtone) nm and 1500 nm (N-H stretch first overtone) have been attributed to proteins [55,60]. When comparing the normalized spectra of both the organically and conventionally produced eggs at the wavelength of 1425 nm, which is related to protein, they were clearly different, indicating that their protein was different. This observation corresponds to the study by Florkiewicz et al. [15], who reported that organic eggs had a higher protein content than conventional eggs.

### 3.4. Discriminant Analysis

**Figure 7:**
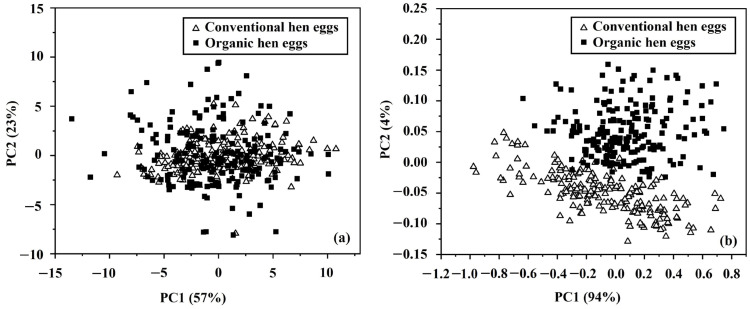
PCA score plots for determination between organic and conventional hens’ eggs by using (**a**) physical properties and (**b**) spectral data.

PCA was used to test the ability of classification between non-destructive techniques by using the physical properties of eggs compared to their spectral data. Independent variables from both techniques were analyzed using PCA to evaluate the accuracy of this classification. The independent variables from physical property measurements were L*, a*, b*, mass, volume, and density, while the independent variables were from the NIR-HSI measurements of spectral data in the wavelength of 935–1720 nm. Figure 7a shows discrimination by using physical property data, with the score plots of the PC1 and PC2. These results showed overlaps between the data from the two production systems of the eggs, but they also showed that PC1 explained 57% of the variance and PC2 explained a further 23% of the variance; hence, the cumulative variance percentage of PC1 and PC2 was 80%. Figure 7b indicates that the score plot of the PC1 and PC2 showed that there was good distinction in the classification between the two types of eggs using spectral data. The variation for PC1 and PC2 was 94% and 4%, respectively, giving the cumulative variance percentage of PC1 and PC2 of 98%. These results therefore imply that using the spectral data of hens’ eggs gave better results than using their physical properties for non-destructively discriminating organically produced eggs from conventionally produced eggs.

**Figure 8:**
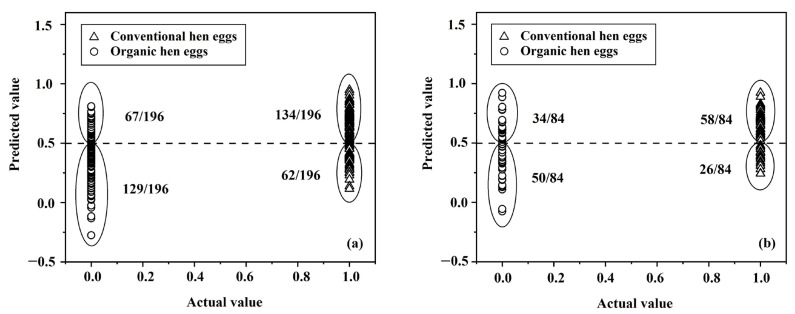
Scatter plots of classification using LDA for organically and conventionally produced eggs in (**a**) the calibration set and (**b**) the prediction set.

The physical properties of eggs were used as independent variables for analyzing the differentiation between conventionally and organically produced eggs using LDA. The results of this classification achieved accuracies of 67.09% (263/392 samples) and 64.29% (108/168 samples) for the calibration and prediction sets, respectively (Table 1). The scatter plots of actual values and predicted values from the classification model using LDA illustrated the differences between conventionally and organically produced eggs in the calibration set and the prediction set (Figure 8).

The average spectral data from spectral images of the eggs, obtained using NIR-HSI, were used to establish the classification models using SVMC and PLS-DA. The spectral data of samples in the calibration set were preprocessed using smoothing, first-derivative, second-derivative, SNV, and MSC methods and combinations of two methods to develop a classification model to differentiate organic eggs from conventional eggs. The best classification models from SVMC and PLS-DA were selected using the optimum results of cross-validation (Table 2). The first derivative combined with the SNV spectral pretreatment method gave the best results for differentiating between the two production systems of eggs using SVMC. For SVMC, the radial bias function (RBF) kernel by Nu and Gamma was used to create the model, as described by Hsu et al. [61]. SVMC was applied by choosing the nu-SVC (nu-support vector classification). The optimal parameters in this analysis were Nu = 0.255 and Gamma = 1. The performance of classification showed accuracy of 97.28% (143/147 samples) for conventional eggs and 96.60% (142/147 samples) for organic eggs. The overall accuracy was 96.94%, with a 3.06% error rate in classification. Meanwhile, the first derivative combined with the MSC spectral pretreatment method gave the best results for classification via PLS-DA. Using the method previously described by Lindstrom et al. [62], who used PLS-DA for multivariate data analysis for classification, the optimal latent variable (LV) for this analysis was 5. The best result showed accuracy of 95.24% (140/147 samples) for conventional eggs and 99.32% (146/147 samples) for organic eggs. The overall accuracy was 97.28%, with a 2.72% error rate in classification. Therefore, PLS-DA showed the best results for classifying between the two types of egg production systems.

**Figure 9:**
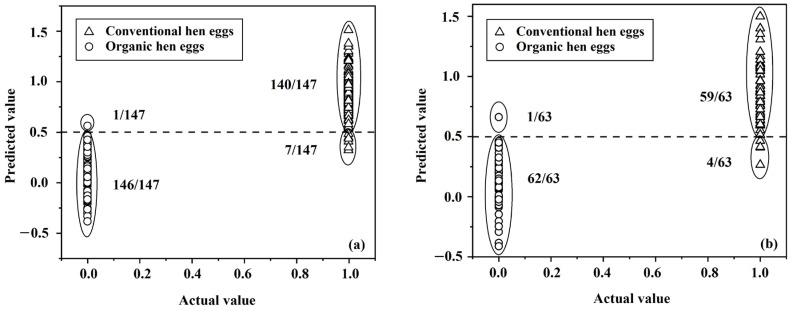
Scatter plots of classification using PLS-DA for organic and conventional hens’ eggs in (**a**) the calibration set and (**b**) the prediction set.

PLS-DA was selected to be used to test the accuracy using the samples in the prediction set in this study. The accuracy of prediction for conventionally produced eggs was 93.65% (59/63 samples). For organically produced eggs, it was 98.41% (62/63 samples), which showed the overall accuracy for prediction of 96.03% and the error rate of 3.97% (Table 3). The accuracy of the scatter plots of actual and predicted values of organic eggs (0) and conventional eggs (1) using PLS-DA (Figure 9) shows the potential NIR-HSI had for determining whether eggs had been produced using organic or conventional production methods.

The results of the classification of eggs, using a statistical comparison, showed that using NIR spectral data for analysis gave higher accuracy than using their physical properties. However, the results showed that the colors of the eggs from the different production methods were significantly different. However, there was a limitation of the NIR-HSI instrument used in this study, which was only able to measure the range of 935–1720 nm. In this regard, it is interesting to pay attention to investigations in the visible wavelength range (380–750 nm) combined with the NIR wavelength and the use of machine learning methods for a further study in order to improve the accuracy in differentiating between conventionally and organically produced eggs.

## 4. Conclusions

The objective of this study was to test whether it could be possible to determine whether an egg had been produced from a hen that had been reared using either organic or conventional production methods. Also, the method needed to be non-destructive, fast, simple, and reliable so that it could be used practically in commercial online grading and verification systems. Therefore, measurements of the physical characteristics and hyperspectral images of samples of eggs were selected and compared. The physical characteristics measured non-destructively from the surface of the eggs can only present external information of eggs, while the light from hyperspectral imaging can penetrate through the shell and therefore can present internal information of eggs. This means that internal information of eggs can possibly be used to differentiate between production systems for eggs more than external information. The results of the principal components analysis obtained using the spectral data from near-infrared hyperspectral imaging measurements of whole hens’ eggs, from organic or conventional production methods, showed that spectral data could be used to non-destructively differentiate whether eggs have been produced using organic or conventional production systems. The spectra of samples were preprocessed using the first derivative combined with multiplicative scatter correction spectral pretreatment, which gave optimal results for establishing the classification model using partial least squares discriminant analysis. The accuracy of prediction for this classification was 96.03%, indicating that it is a possibility that NIR-HSI could be successfully used to differentiate whether an egg has been produced by an organic or conventional production system and thus could be used in a non-destructive online grading system. This system could therefore be useful for food manufacturers for screening raw eggs before production and also to assure consumers that when they pay a premium price for organic eggs, they can be certain they are getting what they pay for.

## Figures and Tables

**Table 1:** The classification results obtained using LDA of the eggs in the calibration set and the prediction set.

Model	DATA SET	Conventional Hen Eggs		Organic Hen Eggs		% Accuracy	% Specificity	% Sensitivity	% Error Rate
		True	False	True	False				
		(TN)	(FN)	(TP)	(FP)				
LDA	Calibration	134/196	62/196	129/196	67/196	67.09	67.54	66.67	32.91
	Prediction	58/84	26/84	50/84	34/84	64.29	65.79	63.04	35.71

**Table 2:** Cross-validation of SVMC and PLS-DA in the calibration set.

Model	Pre-Treatment	Conventional Hen Eggs		Organic Hen Eggs		% Accuracy	% Specificity	% Sensitivity	% Error Rate
		True	False	True	False				
		(TN)	(FN)	(TP)	(FP)				
SVMC	^1^ 1st derivative + ^2^ SNV	143/147	4/147	142/147	5/147	96.94	96.62	97.26	3.06
PLS-DA	1st derivative + ^3^ MSC	140/147	7/147	146/147	1/147	97.28	99.29	95.42	2.72

^1^ 1st derivative = Savitzky–Golay first derivative. ^2^ SNV = standard normal variate transformation. ^3^ MSC = multiplicative scatter correction.

**Table 3:** The results of classification via PLS-DA in the prediction set.

Model	Pre-Treatment	Conventional Hen Eggs		Organic Hen Eggs		% Accuracy	% Specificity	% Sensitivity	%Error Rate
		True	False	True	False				
		(TN)	(FN)	(TP)	(FP)				
PLS-DA	^1^ 1st derivative ^2^ MSC	59/63	4/63	62/63	1/63	96.03	98.33	93.94	3.97

^1^ 1st derivative = Savitzky–Golay first derivative. ^2^ MSC = multiplicative scatter correction.

## Data Availability

The data presented in this study are available on request from the corresponding author.
